# Supplementary material for: Antiglycoxidative Properties of Extracts and Fractions from Reynoutria Rhizomes
Source: Nutrients. 2021 Nov 14;13(11):4066. doi: 10.3390/nu13114066 (PMC8622691; doi:10.3390/nu13114066)
Supplement: Supplementary file 1 [file nutrients-13-04066-s001.zip › nutrients-1444078-supplementary.pdf]

Tukey's multiple comparisons test

* Statistically significant at $p \leq 0.05$, ** for $p \leq 0.01$, *** for $p \leq 0.001$, **** for $p \leq 0.0001$, ns-not statistically significant at $p \leq 0.05$

| | BSA fructosamine | HSA fructosamine | BSA AGE | HSA AGE | BSA carbonyl group | HSA carbonyl group | BSA thiol group | HSA thiol group | BSA Thioflavin T - β amyloid | HSA Thioflavin T - β amyloid | BSA Congo red - β amyloid | HSA Congo red - β amyloid |
|---|---|---|---|---|---|---|---|---|---|---|---|---|
| CONTROL - VS. CONTROL + | **** | **** | **** | **** | **** | **** | **** | **** | **** | **** | **** | **** |
| CONTROL - VS. R.J. ACETONE | **** | **** | **** | **** | ** | ns | **** | **** | **** | ns | **** | ns |
| CONTROL - VS. R.J. DICHLOROMETHANE | **** | **** | **** | **** | **** | **** | **** | **** | **** | ** | **** | **** |
| CONTROL - VS. R.J. DIETHYL ETHER | **** | **** | **** | **** | ns | **** | **** | **** | **** | * | **** | **** |
| CONTROL - VS. R.J. ETHYL ACETATE | **** | **** | **** | **** | **** | ns | **** | **** | **** | *** | **** | ns |
| CONTROL - VS. R.J. BUTANOL | **** | **** | **** | **** | **** | **** | **** | **** | **** | ns | **** | **** |
| CONTROL - VS. R.S. ACETONE | **** | **** | **** | **** | ** | **** | **** | **** | **** | ** | **** | ns |
| CONTROL - VS. R.S. DICHLOROMETHANE | **** | **** | **** | **** | **** | **** | **** | **** | **** | **** | **** | **** |
| CONTROL - VS. R.S. DIETHYL ETHER | **** | **** | **** | **** | **** | **** | **** | **** | **** | ns | **** | ns |
| CONTROL - VS. R.S. ETHYL ACETATE | **** | ns | **** | *** | ns | ** | **** | **** | **** | *** | **** | ns |
| CONTROL - VS. R.S. BUTANOL | **** | **** | **** | **** | **** | **** | **** | **** | **** | ns | **** | ** |

| | | | | | | | | | | | | |
|---|---|---|---|---|---|---|---|---|---|---|---|---|
| CONTROL - VS. R. X B. ACETONE | **** | **** | **** | **** | **** | **** | **** | **** | **** | ns | **** | ns |
| CONTROL - VS. R. X B. DICHLOROMETHANE | **** | **** | **** | **** | **** | **** | **** | **** | ns | ns | **** | **** |
| CONTROL - VS. R. X B. DIETHYL ETHER | **** | **** | **** | **** | **** | **** | **** | **** | **** | ns | **** | ns |
| CONTROL - VS. R. X B. ETHYL ACETATE | **** | **** | **** | ** | ns | ** | **** | **** | **** | **** | ns | ns |
| CONTROL - VS. R. X B. BUTANOL | **** | **** | **** | **** | **** | **** | **** | **** | **** | ns | **** | * |
| CONTROL - VS. RESVERATROL | **** | **** | **** | ns | **** | *** | **** | **** | **** | **** | **** | ns |
| CONTROL - VS. AMINOGUANIDINE | **** | **** | **** | *** | **** | ns | **** | **** | **** | **** | *** | ns |
| CONTROL + VS. R.J. ACETONE | **** | **** | **** | **** | **** | **** | **** | **** | **** | **** | **** | **** |
| CONTROL + VS. R.J. DICHLOROMETHANE | **** | **** | **** | ns | ns | * | ns | *** | **** | **** | **** | Ns |
| CONTROL + VS. R.J. DIETHYL ETHER | **** | **** | **** | **** | **** | **** | **** | **** | **** | **** | **** | ** |
| CONTROL + VS. R.J. ETHYL ACETATE | **** | **** | **** | **** | **** | **** | **** | **** | **** | **** | **** | **** |
| CONTROL + VS. R.J. BUTANOL | **** | *** | **** | ns | **** | **** | **** | ns | **** | **** | **** | ns |
| CONTROL + VS. R.S. ACETONE | **** | **** | **** | **** | **** | **** | **** | **** | **** | **** | **** | **** |
| CONTROL + VS. R.S. DICHLOROMETHANE | **** | **** | **** | * | **** | ns | ns | ns | **** | **** | **** | ns |
| CONTROL + VS. R.S. DIETHYL ETHER | **** | **** | **** | **** | **** | **** | **** | **** | **** | **** | **** | **** |

| | | | | | | | | | | | | |
|---|---|---|---|---|---|---|---|---|---|---|---|---|
| CONTROL + VS. R.S. ETHYL ACETATE | **** | **** | **** | **** | **** | **** | **** | **** | **** | **** | **** | **** |
| CONTROL + VS. R.S. BUTANOL | **** | **** | **** | ** | **** | ns | **** | ns | **** | **** | **** | **** |
| CONTROL + VS. R. X B. ACETONE | **** | **** | **** | **** | **** | **** | **** | **** | **** | **** | **** | **** |
| CONTROL + VS. R. X B. DICHLOROMETHANE | * | ns | **** | ns | **** | ns | ** | ns | **** | **** | **** | **** |
| CONTROL + VS. R. X B. DIETHYL ETHER | **** | **** | **** | ** | **** | **** | **** | **** | **** | **** | **** | **** |
| CONTROL + VS. R. X B. ETHYL ACETATE | **** | **** | **** | **** | **** | **** | **** | **** | **** | **** | **** | **** |
| CONTROL + VS. R. X B. BUTANOL | **** | **** | **** | **** | **** | ns | **** | *** | **** | **** | **** | **** |
| CONTROL + VS. RESVERATROL | **** | **** | **** | **** | **** | **** | **** | **** | **** | **** | **** | **** |
| CONTROL + VS. AMINOGUANIDINE | **** | **** | **** | **** | **** | **** | **** | **** | **** | **** | **** | **** |
| R.J. ACETONE VS. R.J. DICHLOROMETHANE | **** | **** | **** | **** | **** | **** | **** | **** | **** | **** | **** | **** |
| R.J. ACETONE VS. R.J. DIETHYL ETHER | **** | **** | **** | ** | **** | ns | **** | **** | **** | ns | **** | ** |
| R.J. ACETONE VS. R.J. ETHYL ACETATE | **** | **** | **** | ns | ns | ns | **** | **** | **** | ns | **** | ns |
| R.J. ACETONE VS. R.J. BUTANOL | **** | **** | **** | **** | **** | **** | **** | **** | **** | ns | **** | **** |
| R.J. ACETONE VS. R.S. ACETONE | **** | **** | **** | **** | **** | ** | **** | **** | **** | ns | *** | ns |
| R.J. ACETONE VS. R.S. DICHLOROMETHANE | **** | **** | **** | **** | **** | **** | **** | **** | **** | **** | **** | **** |

| | | | | | | | | | | | | |
|---|---|---|---|---|---|---|---|---|---|---|---|---|
| R.J. ACETONE VS. R.S. DIETHYL ETHER | **** | **** | **** | ** | **** | **** | **** | **** | **** | ns | **** | ns |
| R.J. ACETONE VS. R.S. ETHYL ACETATE | **** | **** | **** | **** | ns | ns | **** | **** | **** | ns | **** | ns |
| R.J. ACETONE VS. R.S. BUTANOL | **** | **** | **** | **** | **** | **** | **** | **** | **** | *** | **** | ns |
| R.J. ACETONE VS. R. X B. ACETONE | **** | ns | **** | ns | **** | ns | **** | **** | **** | ns | **** | ns |
| R.J. ACETONE VS. R. X B. DICHLOROMETHANE | **** | **** | **** | **** | **** | **** | **** | **** | **** | ** | **** | * |
| R.J. ACETONE VS. R. X B. DIETHYL ETHER | ns | ns | **** | **** | **** | *** | **** | **** | **** | ns | **** | ns |
| R.J. ACETONE VS. R. X B. ETHYL ACETATE | **** | **** | **** | **** | **** | ns | **** | **** | **** | ns | **** | ns |
| R.J. ACETONE VS. R. X B. BUTANOL | **** | **** | **** | **** | **** | **** | **** | **** | ns | ns | **** | ns |
| R.J. ACETONE VS. RESVERATROL | **** | **** | **** | **** | ns | **** | **** | **** | **** | * | **** | ns |
| R.J. ACETONE VS. AMINOGUANIDINE | **** | **** | **** | **** | ** | **** | **** | * | **** | ns | **** | ns |
| R.J. DICHLOROMETHANE VS. R.J. DIETHYL ETHER | **** | **** | **** | **** | **** | **** | **** | ns | **** | ns | **** | * |
| R.J. DICHLOROMETHANE VS. R.J. ETHYL ACETATE | **** | **** | **** | **** | **** | **** | **** | **** | **** | **** | **** | **** |
| R.J. DICHLOROMETHANE VS. R.J. BUTANOL | **** | **** | **** | ns | **** | ** | **** | ns | **** | *** | **** | ns |

| | | | | | | | | | | | | |
|---|---|---|---|---|---|---|---|---|---|---|---|---|
| R.J. DICHLOROMETHANE VS. R.S. ACETONE | **** | **** | **** | **** | **** | **** | **** | **** | **** | **** | **** | **** |
| R.J. DICHLOROMETHANE VS. R.S. DICHLOROMETHANE | **** | ns | **** | ns | ns | Ns | ns | ** | **** | ns | **** | ns |
| R.J. DICHLOROMETHANE VS. R.S. DIETHYL ETHER | **** | **** | **** | **** | **** | Ns | **** | ns | **** | **** | **** | **** |
| R.J. DICHLOROMETHANE VS. R.S. ETHYL ACETATE | **** | **** | **** | ns | **** | **** | **** | **** | **** | **** | **** | **** |
| R.J. DICHLOROMETHANE VS. R.S. BUTANOL | **** | **** | **** | **** | **** | ** | **** | ns | **** | ns | **** | **** |
| R.J. DICHLOROMETHANE VS. R. X B. ACETONE | **** | **** | **** | **** | **** | **** | **** | **** | **** | **** | **** | **** |
| R.J. DICHLOROMETHANE VS. R. X B. DICHLOROMETHANE | ns | **** | **** | **** | **** | ns | **** | ns | **** | ns | **** | ** |
| R.J. DICHLOROMETHANE VS. R. X B. DIETHYL ETHER | **** | **** | **** | **** | **** | **** | **** | **** | **** | **** | **** | **** |
| R.J. DICHLOROMETHANE | **** | **** | **** | **** | **** | **** | **** | **** | **** | **** | **** | **** |

| | | | | | | | | | | | | |
|---|---|---|---|---|---|---|---|---|---|---|---|---|
| VS. R. X B. ETHYL ACETATE | | | | | | | | | | | | |
| R.J. DICHLOROMETHANE VS. R. X B. BUTANOL | **** | **** | **** | ns | **** | ns | **** | ns | **** | **** | **** | **** |
| R.J. DICHLOROMETHANE VS. RESVERATROL | **** | **** | **** | **** | **** | **** | **** | **** | **** | **** | **** | **** |
| R.J. DICHLOROMETHANE VS. AMINOGUANIDINE | **** | **** | **** | **** | **** | **** | **** | **** | **** | **** | **** | **** |
| R.J. DIETHYL ETHER VS. R.J. ETHYL ACETATE | **** | **** | **** | **** | **** | ** | **** | **** | **** | **** | **** | *** |
| R.J. DIETHYL ETHER VS. R.J. BUTANOL | ** | **** | **** | **** | **** | ** | **** | **** | **** | ** | **** | ns |
| R.J. DIETHYL ETHER VS. R.S. ACETONE | **** | **** | **** | **** | * | ns | **** | **** | **** | **** | **** | **** |
| R.J. DIETHYL ETHER VS. R.S. DICHLOROMETHANE | **** | **** | **** | **** | **** | **** | **** | **** | **** | ns | *** | ns |
| R.J. DIETHYL ETHER VS. R.S. DIETHYL ETHER | **** | **** | **** | **** | **** | **** | **** | ns | **** | **** | **** | * |
| R.J. DIETHYL ETHER VS. R.S. ETHYL ACETATE | **** | **** | **** | **** | * | ns | **** | **** | **** | **** | **** | *** |
| R.J. DIETHYL ETHER VS. R.S. BUTANOL | **** | **** | **** | **** | **** | **** | **** | **** | **** | ns | **** | ns |

| | | | | | | | | | | | | |
|---|---|---|---|---|---|---|---|---|---|---|---|---|
| R.J. DIETHYL ETHER VS. R. X B. ACETONE | **** | **** | **** | **** | **** | ns | **** | **** | **** | **** | **** | **** |
| R.J. DIETHYL ETHER VS. R. X B. DICHLOROMETHANE | **** | **** | **** | **** | **** | **** | **** | **** | **** | ns | **** | ns |
| R.J. DIETHYL ETHER VS. R. X B. DIETHYL ETHER | **** | **** | **** | **** | **** | ns | **** | ns | **** | **** | **** | **** |
| R.J. DIETHYL ETHER VS. R. X B. ETHYL ACETATE | **** | **** | **** | **** | ns | ns | **** | **** | **** | **** | **** | **** |
| R.J. DIETHYL ETHER VS. R. X B. BUTANOL | ns | ** | **** | ns | **** | **** | **** | * | **** | **** | **** | ns |
| R.J. DIETHYL ETHER VS. RESVERATROL | **** | **** | **** | **** | **** | **** | **** | **** | **** | **** | **** | **** |
| R.J. DIETHYL ETHER VS. AMINOGUANIDINE | **** | **** | **** | **** | **** | **** | **** | **** | **** | **** | **** | **** |
| R.J. ETHYL ACETATE VS. R.J. BUTANOL | **** | **** | **** | **** | **** | **** | **** | **** | **** | ** | **** | ** |
| R.J. ETHYL ACETATE VS. R.S. ACETONE | **** | **** | **** | * | **** | **** | **** | **** | ns | ns | **** | ns |
| R.J. ETHYL ACETATE VS. R.S. DICHLOROMETHANE | ns | **** | **** | **** | **** | **** | **** | **** | **** | **** | **** | ** |
| R.J. ETHYL ACETATE VS. R.S. DIETHYL ETHER | **** | **** | **** | ns | **** | **** | **** | **** | ns | ns | **** | ns |
| R.J. ETHYL ACETATE VS. R.S. ETHYL ACETATE | **** | **** | **** | * | ns | ns | **** | **** | ns | ns | **** | ns |

| | | | | | | | | | | | | |
|---|---|---|---|---|---|---|---|---|---|---|---|---|
| R.J. ETHYL ACETATE VS. R.S. BUTANOL | ** | **** | **** | **** | **** | **** | **** | **** | **** | **** | **** | ns |
| R.J. ETHYL ACETATE VS. R. X B. ACETONE | ns | **** | **** | ns | **** | *** | **** | **** | **** | ns | **** | ns |
| R.J. ETHYL ACETATE VS. R. X B. DICHLOROMETHANE | **** | **** | **** | **** | **** | **** | **** | **** | **** | **** | **** | ns |
| R.J. ETHYL ACETATE VS. R. X B. DIETHYL ETHER | ns | **** | **** | **** | **** | **** | **** | **** | **** | ns | **** | ns |
| R.J. ETHYL ACETATE VS. R. X B. ETHYL ACETATE | **** | ns | **** | ** | **** | ns | **** | **** | **** | ns | **** | ** |
| R.J. ETHYL ACETATE VS. R. X B. BUTANOL | **** | **** | **** | **** | **** | **** | **** | **** | **** | ns | **** | ns |
| R.J. ETHYL ACETATE VS. RESVERATROL | **** | **** | **** | **** | ns | **** | **** | **** | **** | ns | **** | ns |
| R.J. ETHYL ACETATE VS. AMINOGUANIDINE | **** | **** | **** | ** | ns | ns | **** | **** | **** | ns | **** | * |
| R.J. BUTANOL VS. R.S. ACETONE | **** | **** | **** | **** | **** | ns | **** | **** | **** | ns | **** | **** |
| R.J. BUTANOL VS. R.S. DICHLOROMETHANE | **** | **** | **** | ** | * | **** | **** | ns | **** | **** | **** | ns |
| R.J. BUTANOL VS. R.S. DIETHYL ETHER | **** | **** | **** | **** | **** | ns | ns | ns | **** | ns | **** | ** |
| R.J. BUTANOL VS. R.S. ETHYL ACETATE | **** | **** | **** | **** | **** | **** | **** | **** | **** | ** | **** | **** |
| R.J. BUTANOL VS. R.S. BUTANOL | **** | *** | **** | * | ns | **** | ** | ns | **** | ns | **** | ns |

| | | | | | | | | | | | | |
|---|---|---|---|---|---|---|---|---|---|---|---|---|
| R.J. BUTANOL VS. R. X B. ACETONE | **** | **** | **** | **** | **** | * | **** | **** | **** | ns | **** | **** |
| R.J. BUTANOL VS. R. X B. DICHLOROMETHANE | **** | **** | **** | ns | **** | **** | * | ns | **** | ns | **** | ns |
| R.J. BUTANOL VS. R. X B. DIETHYL ETHER | **** | **** | **** | * | **** | ns | **** | **** | **** | ns | **** | **** |
| R.J. BUTANOL VS. R. X B. ETHYL ACETATE | **** | **** | **** | **** | **** | **** | **** | **** | **** | **** | **** | **** |
| R.J. BUTANOL VS. R. X B. BUTANOL | ** | **** | **** | **** | **** | **** | ns | ns | **** | ns | **** | * |
| R.J. BUTANOL VS. RESVERATROL | **** | **** | **** | **** | **** | **** | **** | **** | **** | **** | **** | **** |
| R.J. BUTANOL VS. AMINOGUANIDINE | **** | **** | **** | **** | **** | **** | **** | **** | **** | *** | **** | **** |
| R.S. ACETONE VS. R.S. DICHLOROMETHANE | **** | **** | **** | **** | **** | **** | **** | **** | **** | **** | **** | **** |
| R.S. ACETONE VS. R.S. DIETHYL ETHER | ns | **** | **** | ns | ns | * | **** | **** | ns | ns | **** | ns |
| R.S. ACETONE VS. R.S. ETHYL ACETATE | **** | **** | ns | ns | **** | ns | **** | **** | ns | ns | * | ns |
| R.S. ACETONE VS. R.S. BUTANOL | **** | **** | **** | **** | **** | **** | ns | **** | **** | **** | **** | ns |
| R.S. ACETONE VS. R. X B. ACETONE | **** | **** | **** | * | ns | ns | **** | **** | **** | ns | **** | ns |
| R.S. ACETONE VS. R. X B. DICHLOROMETHANE | **** | **** | **** | **** | **** | **** | **** | **** | **** | **** | **** | * |
| R.S. ACETONE VS. R. X B. DIETHYL ETHER | **** | **** | **** | **** | ns | ns | **** | *** | **** | ns | **** | ns |
| R.S. ACETONE VS. R. X B. ETHYL ACETATE | ns | *** | ** | ns | ns | ns | ns | **** | **** | ns | **** | ns |

| | | | | | | | | | | | | |
|---|---|---|---|---|---|---|---|---|---|---|---|---|
| R.S. ACETONE VS. R. X B. BUTANOL | **** | **** | **** | **** | * | **** | **** | **** | **** | ns | **** | ns |
| R.S. ACETONE VS. RESVERATROL | **** | **** | **** | ns | **** | **** | **** | **** | **** | ns | **** | ns |
| R.S. ACETONE VS. AMINOGUANIDINE | ns | ns | * | ns | **** | **** | **** | **** | **** | ns | **** | ns |
| R.S. DICHLOROMETHANE VS. R.S. DIETHYL ETHER | **** | **** | **** | **** | **** | ** | *** | *** | **** | **** | **** | ** |
| R.S. DICHLOROMETHANE VS. R.S. ETHYL ACETATE | **** | **** | **** | **** | **** | **** | **** | **** | **** | **** | **** | **** |
| R.S. DICHLOROMETHANE VS. R.S. BUTANOL | ns | **** | **** | **** | **** | ns | **** | ns | **** | *** | **** | ns |
| R.S. DICHLOROMETHANE VS. R. X B. ACETONE | ns | **** | **** | **** | **** | **** | **** | **** | **** | **** | **** | **** |
| R.S. DICHLOROMETHANE VS. R. X B. DICHLOROMETHANE | **** | **** | **** | *** | **** | ns | ns | ns | **** | **** | **** | ns |
| R.S. DICHLOROMETHANE VS. R. X B. DIETHYL ETHER | * | **** | **** | **** | **** | **** | **** | **** | **** | **** | **** | **** |
| R.S. DICHLOROMETHANE VS. R. X B. ETHYL ACETATE | **** | **** | **** | **** | **** | **** | **** | **** | **** | **** | **** | **** |

| | | | | | | | | | | | | |
|---|---|---|---|---|---|---|---|---|---|---|---|---|
| R.S. DICHLOROMETHANE VS. R. X B. BUTANOL | **** | **** | **** | **** | **** | ns | **** | ** | **** | **** | **** | * |
| R.S. DICHLOROMETHANE VS. RESVERATROL | **** | **** | **** | **** | **** | **** | **** | **** | **** | **** | **** | **** |
| R.S. DICHLOROMETHANE VS. AMINOGUANIDINE | **** | **** | **** | **** | **** | **** | **** | **** | **** | **** | **** | **** |
| R.S. DIETHYL ETHER VS. R.S. ETHYL ACETATE | **** | **** | **** | ns | **** | **** | **** | **** | ns | ns | **** | ns |
| R.S. DIETHYL ETHER VS. R.S. BUTANOL | **** | **** | **** | **** | **** | **** | **** | ns | **** | **** | **** | ns |
| R.S. DIETHYL ETHER VS. R. X B. ACETONE | **** | **** | **** | ns | ns | *** | **** | **** | ** | ns | **** | ns |
| R.S. DIETHYL ETHER VS. R. X B. DICHLOROMETHANE | **** | **** | **** | **** | ** | **** | ns | ns | **** | ** | **** | ns |
| R.S. DIETHYL ETHER VS. R. X B. DIETHYL ETHER | **** | **** | **** | **** | ns | ns | **** | *** | **** | ns | **** | ns |
| R.S. DIETHYL ETHER VS. R. X B. ETHYL ACETATE | ns | **** | ** | ns | ** | **** | **** | **** | **** | ns | **** | * |
| R.S. DIETHYL ETHER VS. R. X B. BUTANOL | **** | **** | **** | **** | ns | * | ns | ns | **** | ns | **** | ns |
| R.S. DIETHYL ETHER VS. RESVERATROL | **** | **** | **** | ** | **** | **** | **** | **** | **** | ns | **** | ns |
| R.S. DIETHYL ETHER VS. AMINOGUANIDINE | ns | **** | **** | ns | **** | **** | **** | **** | **** | ns | **** | ns |

| | | | | | | | | | | | | |
|---|---|---|---|---|---|---|---|---|---|---|---|---|
| R.S. ETHYL ACETATE VS. R.S. BUTANOL | **** | **** | **** | **** | **** | **** | **** | **** | **** | **** | **** | ns |
| R.S. ETHYL ACETATE VS. R. X B. ACETONE | **** | **** | **** | * | **** | ns | **** | **** | **** | ns | **** | ns |
| R.S. ETHYL ACETATE VS. R. X B. DICHLOROMETHANE | **** | **** | **** | **** | **** | **** | **** | **** | **** | **** | **** | ** |
| R.S. ETHYL ACETATE VS. R. X B. DIETHYL ETHER | **** | **** | **** | **** | **** | * | **** | **** | **** | ns | *** | ns |
| R.S. ETHYL ACETATE VS. R. X B. ETHYL ACETATE | **** | **** | **** | ns | **** | ns | ** | ** | **** | ns | **** | ns |
| R.S. ETHYL ACETATE VS. R. X B. BUTANOL | **** | **** | **** | **** | **** | **** | **** | **** | **** | ns | **** | ns |
| R.S. ETHYL ACETATE VS. RESVERATROL | ns | ** | **** | ns | * | **** | **** | **** | **** | ns | **** | ns |
| R.S. ETHYL ACETATE VS. AMINOGUANIDINE | **** | **** | ns | ns | **** | **** | **** | **** | **** | ns | **** | ns |
| R.S. BUTANOL VS. R. X B. ACETONE | ns | **** | **** | **** | **** | **** | **** | **** | **** | **** | **** | * |
| R.S. BUTANOL VS. R. X B. DICHLOROMETHANE | **** | **** | **** | ns | ns | ns | **** | ns | **** | ns | **** | ns |
| R.S. BUTANOL VS. R. X B. DIETHYL ETHER | **** | **** | ns | ns | **** | **** | **** | **** | **** | ** | **** | ns |
| R.S. BUTANOL VS. R. X B. ETHYL ACETATE | **** | **** | **** | **** | **** | **** | * | **** | **** | **** | **** | **** |
| R.S. BUTANOL VS. R. X B. BUTANOL | **** | ns | **** | * | *** | ns | **** | ns | **** | *** | **** | ns |
| R.S. BUTANOL VS. RESVERATROL | **** | **** | **** | **** | **** | **** | **** | **** | **** | **** | **** | ** |

| | | | | | | | | | | | | |
|---|---|---|---|---|---|---|---|---|---|---|---|---|
| R.S. BUTANOL VS. AMINOGUANIDINE | **** | **** | **** | **** | **** | **** | **** | **** | **** | **** | **** | *** |
| R. X B. ACETONE VS. R. X B. DICHLOROMETHANE | **** | **** | **** | **** | * | **** | **** | **** | **** | *** | **** | *** |
| R. X B. ACETONE VS. R. X B. DIETHYL ETHER | ** | ns | **** | **** | ** | ns | **** | **** | **** | ns | ns | ns |
| R. X B. ACETONE VS. R. X B. ETHYL ACETATE | **** | **** | **** | ** | ns | ns | **** | ns | **** | ns | **** | ns |
| R. X B. ACETONE VS. R. X B. BUTANOL | **** | **** | **** | **** | ns | **** | **** | **** | ** | ns | **** | ns |
| R. X B. ACETONE VS. RESVERATROL | **** | **** | **** | **** | **** | **** | **** | **** | **** | ns | **** | ns |
| R. X B. ACETONE VS. AMINOGUANIDINE | **** | **** | **** | ** | **** | **** | **** | **** | **** | ns | **** | ns |
| R. X B. DICHLOROMETHANE VS. R. X B. DIETHYL ETHER | **** | **** | **** | ns | * | **** | **** | **** | **** | * | **** | *** |
| R. X B. DICHLOROMETHANE VS. R. X B. ETHYL ACETATE | **** | **** | **** | **** | **** | **** | **** | **** | **** | **** | **** | **** |
| R. X B. DICHLOROMETHANE VS. R. X B. BUTANOL | **** | **** | **** | **** | ns | ns | ns | ns | **** | * | **** | ns |
| R. X B. DICHLOROMETHANE VS. RESVERATROL | **** | **** | **** | **** | **** | **** | **** | **** | **** | **** | **** | **** |
| R. X B. DICHLOROMETHANE | **** | **** | **** | **** | **** | **** | **** | **** | **** | **** | **** | **** |

| | | | | | | | | | | | | |
|---|---|---|---|---|---|---|---|---|---|---|---|---|
| VS. AMINOGUANIDINE | | | | | | | | | | | | |
| R. X B. DIETHYL ETHER VS. R. X B. ETHYL ACETATE | **** | **** | **** | **** | ** | ns | **** | **** | **** | * | **** | ns |
| R. X B. DIETHYL ETHER VS. R. X B. BUTANOL | **** | **** | **** | * | ns | **** | **** | **** | **** | ns | **** | ns |
| R. X B. DIETHYL ETHER VS. RESVERATROL | **** | **** | **** | **** | **** | **** | **** | **** | **** | ** | **** | ns |
| R. X B. DIETHYL ETHER VS. AMINOGUANIDINE | **** | **** | **** | **** | **** | **** | **** | **** | **** | ns | **** | ns |
| R. X B. ETHYL ACETATE VS. R. X B. BUTANOL | **** | **** | **** | **** | **** | **** | **** | **** | **** | * | **** | *** |
| R. X B. ETHYL ACETATE VS. RESVERATROL | **** | **** | **** | ns | **** | **** | **** | **** | ** | ns | **** | ns |
| R. X B. ETHYL ACETATE VS. AMINOGUANIDINE | ns | **** | **** | ns | **** | **** | **** | **** | ns | ns | ns | ns |
| R. X B. BUTANOL VS. RESVERATROL | **** | **** | **** | **** | **** | **** | **** | **** | **** | ** | **** | * |
| R. X B. BUTANOL VS. AMINOGUANIDINE | **** | **** | **** | **** | **** | **** | **** | **** | **** | ns | **** | ** |
| RESVERATROL VS. AMINOGUANIDINE | **** | **** | **** | ns | **** | ns | **** | **** | ** | ns | **** | ns |
